# Supplementary material for: Glyphosate and its formulations Roundup Bioflow and RangerPro alter bacterial and fungal community composition in the rat caecum microbiome
Source: Front Microbiol. 2022 Oct 5;13:888853. doi: 10.3389/fmicb.2022.888853 (PMC9580462; doi:10.3389/fmicb.2022.888853)
Supplement: Supplementary file 5 [file Presentation_1.pdf]

Supplementary Material

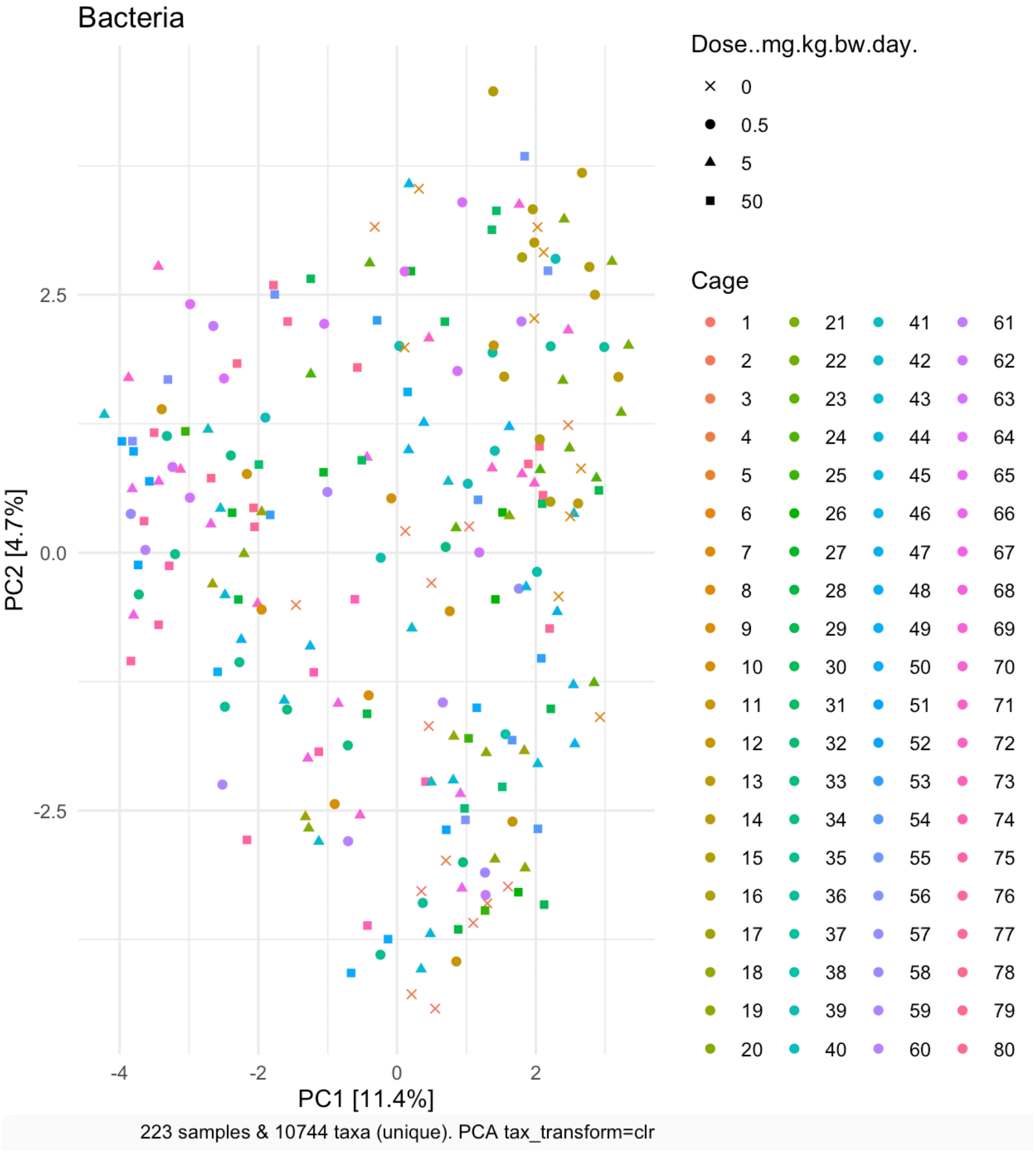

Supplementary Figure 1. Unsupervised classification of variations in bacterial community composition by PCA for the cage effects. Male and female Sprague Dawley rats were exposed to three doses of glyphosate (0.5, 5, 50 mg/kg body weight per day), or to the formulated products Roundup Bioflow and RangerPro at the same glyphosate-equivalent doses starting at mid-gestation and ending at 13 weeks post-weaning. The PCA was calculated using centred log-ratio transformed abundance data from bacterial 16S rRNA amplicon sequencing of the caecum microbiota.

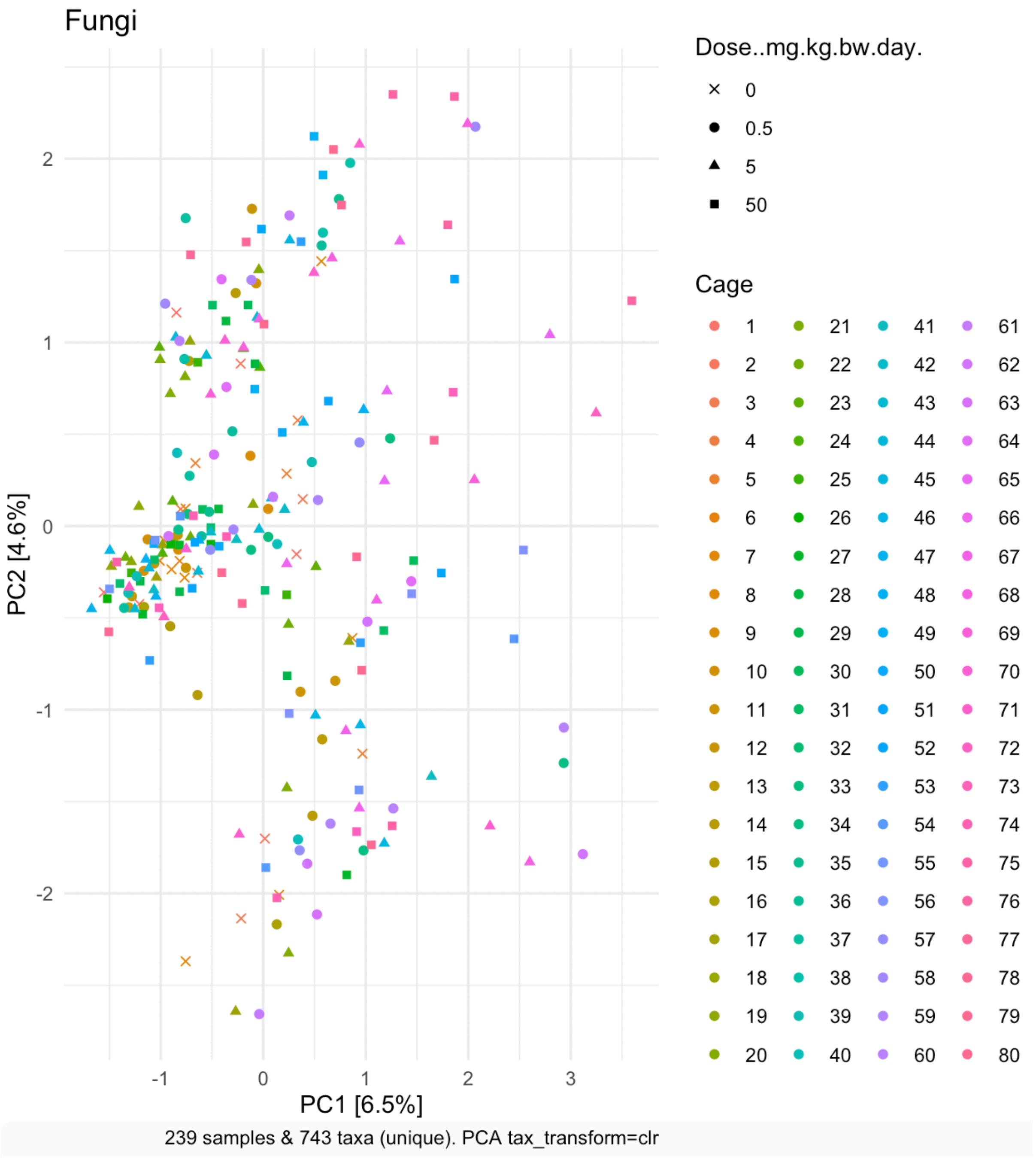

Supplementary Figure 2. Unsupervised classification of variations in fungal community composition by PCA for the cage effects. Male and female Sprague Dawley rats were exposed to three doses of glyphosate (0.5, 5, 50 mg/kg body weight per day), or to the formulated products Roundup Bioflow and RangerPro at the same glyphosate-equivalent doses starting at mid-gestation and ending at 13 weeks post-weaning. The PCA was calculated using centred log-ratio transformed abundance data from fungal ITS2 amplicon sequencing of the caecum microbiota.
